# Springback Reduction of Ultra-High-Strength Martensitic Steel Sheet by Electrically Single-Pulsed Current

**DOI:** 10.3390/ma15072373

**Published:** 2022-03-23

**Authors:** Minki Kim, Gihyun Bae, Namsu Park, Jung Han Song

**Affiliations:** Molding & Metal Forming R&D Department, Korea Institute of Industrial Technology (KITECH), 156, Gaetbeol-ro, Yeonsu-gu, Incheon 21999, Korea; mkim@kitech.re.kr (M.K.); baegh@kitech.re.kr (G.B.); nspark@kitech.re.kr (N.P.)

**Keywords:** springback, ultra-high-strength steel, V-bending, single-pulsed current, electric energy density, electro-plasticity

## Abstract

This paper investigates the reduction of springback by an electrically single-pulsed current for an ultra-high-strength martensitic steel sheet, MART1470 1.2t. In order to evaluate the springback reduction by the electric current, V-bending tests were performed with various parameter-sets (current density and pulse duration). The amount of springback reduction was then calculated from the measured bent-angle of tested specimens. Experimental results show the springback is reduced with the increase in the current density, the pulse duration, and the electric energy density. In order to clarify thermal and athermal portions in the effect of electric current on the springback reduction, two ratios of force and isothermal flow stress were calculated based on bending theory. From the comparison of the ratios, it is noted that the athermal portion mainly contributes to the force relaxation, so the springback amount decreases. The athermal portion significantly increases as the electric energy density increases. Microstructures and micro-Vickers hardness were observed to confirm the applicability of the single-pulsed current to forming processes in practice. The springback reduction can be achieved up to 37.5% without severe changes in material properties when the electric energy density increases up to 281.3 mJ/mm^3^. Achievable reduction is 85.4% for the electric energy density of 500 mJ/mm^3^, but properties remarkably change.

## 1. Introduction

The development of structural materials is being required more and more to achieve the lightweight design with enhanced stability and crashworthiness of structures. Ultra-high-strength steel (UHSS) is introduced to provide a good candidate for the structural material. A challenge of using high-strength steel is to overcome the springback issue for attaining net-shaped products after the sheet metal forming processes [1]. Especially for ultra-high-strength martensitic steels, the amount of springback is significant because of high flow stress and stiffness [2]. Numerous research works have been performed to enhance traditional approaches to compensate for the springback. From a process point of view, stress control or rebalancing is realized by changing processes, including over-forming [3] and designs of tools and dies [4]. Warm or hot stamping [5] is utilized to reduce the springback by controlling the flow stress level and stiffness. For a better understanding of the springback phenomenon in a forming process, the relationship between the springback and material behavior has been continuously identified through performing lab-scaled bending tests such as L- [6], U- [7], and V-bending [8]. In hot or warm forming, residual stress [9] and stress relaxation due to holding time [10] are representative variables to reduce the springback. Since effects of strain rate and asymmetry on flow stress levels are significant [11], further research on the springback has been conducted to address advanced consideration of variables in forming process [12,13].

Since electro-plasticity [14] was recently reported, the effect of electric currents on material behavior has been focused on developing new approaches for metal forming processes, so-called electrically assisted forming (EAF) [15]. The electro-plasticity introduces that the plasticity increases when the electric current is applied to materials due to the electro-migration [16], temperature rising from the Joule heating [17], the microstructure evolution [18], and so on. Applying the electric current to materials induces both thermal and athermal effects, which are temperature effects from the Joule heating and changes in microstructural behavior by the electric current, respectively. It is, however, hard to determine the dominance of each because it depends on the material [8,19]. The electric current is classified into two concepts, continuous and pulsed (discontinuous) currents. The pulsed current with a proper parameter-set (current density, pulse duration, and pulse period) is effective to enhance the formability without severe temperature rising from the Joule heating that could change the microstructure of a metal [20,21]. 

To reduce the springback, a combination of the electric current and the traditional forming processes would be a cost-efficient approach because stress level distributed in the workpiece can be reduced due to rapid heating or the electro-plasticity during the forming process at room temperature, while the traditional hot or warm forming needs relatively slow and overall heating procedures with a thermal-environmental chamber. The majority of research works regarding the electric current on material behavior is observed the effect of electric current during the deformation. However, the number of research works related to the electric current effect on post-deformation behavior is very limited to identifying the effect on the springback. Applying the electric current to the material after deformation would be a good opportunity to reduce the springback with a simple procedure compared to in situ application of electric current. 

In this paper, the simplest one of electrically pulsed current, i.e., single-pulsed current, is selected to be applied to an ultra-high-strength martensitic steel sheet of MART1470 1.2t. The present study is related to an extended work with more systematic approaches for a different UHSS from the author’s previous work [8]. In order to investigate the effect of the single-pulsed current on the springback reduction, V-bending experiments are performed. Currents with various parameter-sets (current density and pulse duration) are then applied to specimens after the V-bending tests. The amounts of the springback with respect to the electric energy are evaluated to identify the effect of electrically single-pulsed current on the springback reduction. In order to confirm the applicability of the single-pulsed current to the sheet metal forming process in practice, changes to the hardness and the microstructure are observed to investigate whether the springback can be reduced without severe changes in material properties due to the electric current or not.

## 2. Materials and Methods

An ultra-high-strength martensitic steel sheet of MART1470 (1.2 mm thickness) is selected to investigate the effect of electrically single-pulsed current after V-bending tests. The as-received material is a cold-rolled and uncoated one priorly experienced hot-rolling with quenching to transform austenite to martensite and cooling in annealing line during the production process. The martensitic steel is composed of martensite matrix with a small amount of bainite or ferrite. The chemical composition of the MART1470 sheet is listed in Table 1. The MART1470 is designed to obtain a yield strength higher than 1050 MPa, an ultimate tensile strength (UTS) higher than 1470 MPa, and about 9% total elongation. The martensitic steel is applied to manufacturing of vehicle components for good crashworthiness, for example, side sill outer, bumper beam, and outer frame of battery packs. Table 2 provides parameters of the Swift hardening equation, which is widely used for steel sheets. The parameters were obtained by fitting uniaxial tension data at the static state and the room temperature. Details of the obtained uniaxial tension data will be addressed in Section 4. Since the MART1470 has very high strength, the springback issue after sheet metal forming processes is significant. In this paper, the MART1470 was tested with a V-bending system under an electric current condition to evaluate the amount of springback.

### 2.1. Experimental Set-Up

**Figure 1:**
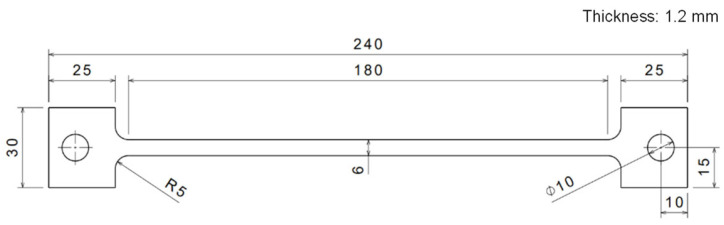
Specimen dimensions (unit: mm).

In order to confirm the amount of springback, test specimens were prepared from the sheet with a large-scaled dog-bone shape, as shown in Figure 1. Each specimen has a rectangular test section with 180 mm length and 6 mm width. The length of specimen was aligned with the rolling direction (RD) of the sheet. Two grip sections of each specimen exist to connect electrodes of a power supply for imposing the electric current. All specimens were blotted and polished by using the oil paper and a 2000 grit abrasive paper to remove oil-layer and contamination on the surface because those would lead to being locally burnt with sparks while the electric current is applying.

**Figure 2:**
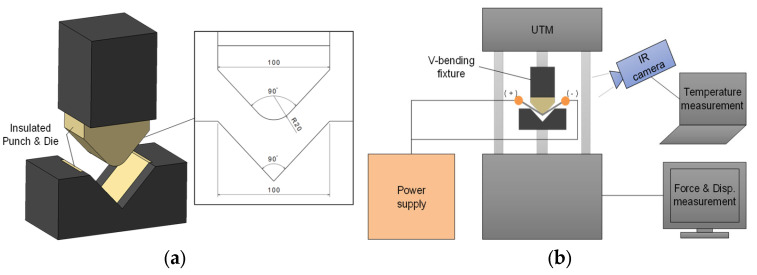
Experimental set-up: (**a**) V-bending fixtures (unit: mm); (**b**) Schematic diagram of overall set-up.

V-bending testing was designed to perform lab-scaled springback tests, as shown in Figure 2. In Figure 2a, the punch and the die were developed to be assembled into a universal testing machine (UTM). With consideration of applying the electric current, the fixtures were fabricated with a machinable glass-ceramic MACOR^®^ (CORNING Inc., Corning, NY, USA) to insulate themselves from the current. The V-shaped punch and die adopted a bent-angle of 90°, a punch radius of 20 mm, and a die width of 100 mm. Figure 2b illustrates a schematic diagram of overall V-bending tests with the electric current and measurement systems. The bending tests were carried out by using the MTS810 (MTS Systems Corporation, Eden Prairie, MN, USA), which is a conventional UTM. For generating the electric current, a power supply SPU-1000 (Hyundai Welding, Seoul, South Korea) was utilized. The anode and cathode electrodes were installed to each grip section of a specimen, respectively. Force and displacement data were obtained from the UTM while temperature of specimen was measured from an infra-red (IR) camera system, FLIR T620 (Teledyne FLIR, Wilsonville, OR, USA), to observe temperature changes of a specimen due to the Joule heating.

### 2.2. V-Bending Test with Single-Pulsed Current

**Figure 3:**
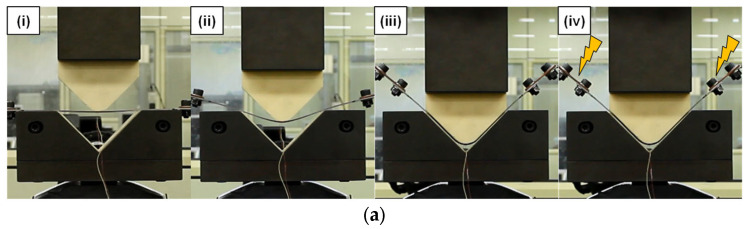

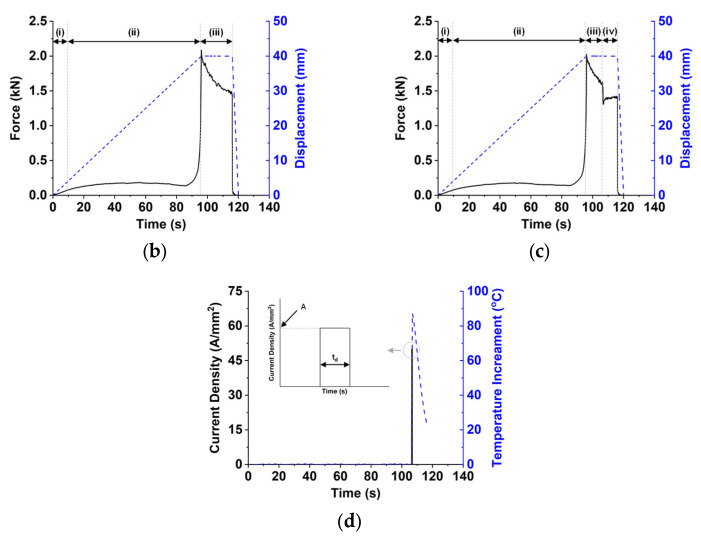
V-bending test: (**a**) Procedure; (**b**) Force and displacement during test without the electric current; (**c**) Force and displacement during test with the electric current; (**d**) Single-pulsed current and temperature increment.

V-bending tests were performed with the punch speed of 25 mm/min as shown in Figure 3. Figure 3a shows the procedure of the V-bending with the electric current. Four steps are (i) The punch partially contacts with the specimen; (ii) The punch fully contacts with the specimen and the specimen are bent; (iii) The punch stops for 10 s (holding) when the displacement reaches a certain amount that the gap between the punch and die is the same with the specimen thickness; (iv) The single-pulsed current is applied to the specimen while the punch keeps stating the position for 10 s. After the steps, the specimen was unloaded by moving the punch up. Figure 3b,c represent examples of the force or the displacement with respect to the time during the V-bending tests without and with the electric current, respectively. In both cases of tests with and without the electric current, total holding time was 20 s. The holding time in the case of test without the electric current was 20 s instead of 10 s in step (iii). For the test with the electric current, however, the electric current was applied after 10 s of holding at the step (iv). During the holding, force relaxation was observed in both cases. Especially for the case of test with the electric current, the force level was suddenly dropped when the electric current was applied. The force was then increased since the electric current was released. Figure 3d shows examples of the single-pulsed current applied and temperature increment from the Joule heating for the case of test with the electric current. The single-pulsed current has two parameters of nominal current density (A) and pulse duration (t_d_). The nominal current density is defined with the current value divided by the initial area of the specimen (thickness times width). Temperature of a specimen increased when the electric current was applied while rapidly decreasing when the current was released from the specimen.

In order to identify the effect of the electric current in detail, various conditions of the single-pulsed current were applied to specimens with different current densities and pulse durations, as listed in Table 3. For the effect of the current density, the pulse duration was fixed with 0.50 s while the nominal current densities were 50, 75, and 100 A/mm^2^. On the other hand, related to the effect of the pulse duration, the current density was fixed with 75 A/mm^2^ while the pulse durations were 0.25, 0.50, and 0.75 s. The single-pulsed current was generated from the power supply with a square shape, so the electric current consistently applied to the specimen for the pulse duration. All tests were performed three times to acquire repeatability.

### 2.3. Measurement of Springback Angle

Although the bent-angle of the specimen during the holding is 90°, the resultant angle after the unloading is larger than 90° because of the springback. After four steps and unloading in the V-bending test, the specimen was detached from the fixtures. The bent-angle of the specimen (θ (°)) was then measured by using a 3D scan-measurement, the Mitutoyo APEX S500 (Mitutoyo, Kawasaki, Japan). The APEX S500 has the maximum permissible error of 1.7 μm which would be small enough to precisely measure profile of the specimen compared to the gauge length of the specimen (180 mm). It is, therefore, possible to obtain a reliable bent-angle calculated from the profile by the software (Geopak) corresponding to the APEX S500. The springback angle is defined as Equation (1).

(1) Springback angle (°)=θ−90

Springback angles of all specimens with and without the electrically single-pulsed currents were measured to evaluate the effect of current on the springback.

## 3. Results

A parametric study with the V-shaped fixtures was carried out with different single-pulsed currents. From the experimental results of the springback measurements, springback reduction by various single-pulsed currents is addressed in this section. The amount of the springback reduction is defined as Equation (2) where $\theta_{\mathrm{ref}}$ (°) is the bent-angle without the electric current and θ (°) is the bent-angle with the electric current.

(2) $$\mathrm{Springback}\;\mathrm{Reduction}\;(\%)=\frac{\theta_{\mathrm{ref}}-\theta }{\theta_{\mathrm{ref}}-90}\times 100$$

**Figure 4:**
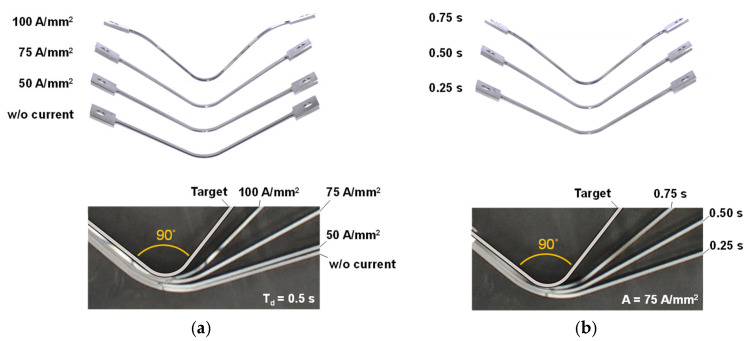
Deformed specimens after tests with different: (**a**) Current densities; (**b**) Pulse durations.

**Figure 5:**
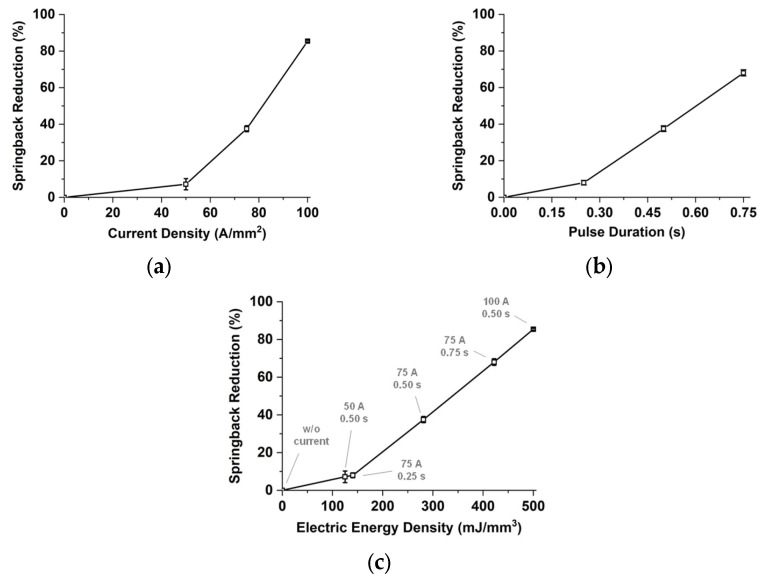
Springback reduction regarding: (**a**) Current density; (**b**) Pulse duration; (**c**) Electric energy density.

Figure 4 shows the deformed specimens after the V-bending tests. For the springback evaluation, all measured data are listed in Table 4. In the springback angle and reduction columns, values of the standard deviations with “±” next to the average values for the repeatability. Side views of the deformed specimens with different current densities and the pulse durations are demonstrated in Figure 4a,b, respectively. As shown in Figure 4a, the springback of the MART1470 sheet is significant without the electric current. The bent-angle of the deformed specimens becomes close to the target angle of 90° as the nominal current density increases. In the case of 50 A/mm^2^, the deformed shape is almost the same as that without the electric current. However, the shape of the specimen is very close to the target shape when the current density is 100 A/mm^2^. The springback angle decreases from 31.61° to 4.96° with an increase in the current density. Regarding the pulse duration in Figure 4b, the bent-angle of the deformed specimens becomes close to the target angle as the pulse duration increases. The springback angle decreases up to 10.89° when the pulse duration increases. Figure 5 represents the springback reduction under various conditions of the electrically single-pulsed current. The error bars indicate the standard deviation for springback reduction of tested specimens. The springback amount is reduced by controlling the current density from 7.2% to 85.4%, as shown in Figure 5a. It is decreased from 9.0% to 68.0% by an increase in the pulse duration, as shown in Figure 5b. In order to compare the effect of parameters consistently, the electric energy density of each condition is calculated by Equation (3) [20].

(3) $$\mathrm{Electric}\;\mathrm{Energy}\;\mathrm{Density}\;(\mathrm{mJ}/{\mathrm{mm}}^{3})=\frac{I^{2}{\mathrm{Rt}}_{d}}{V_{0}}=\frac{I^{2}}{A_{0}l_{0}}\frac{{\rho l}_{0}}{A_{0}}t_{d}={\left(\frac{I}{A_{0}}\right)}^{2}{\rho t}_{d}$$

Equation (3) contains the initial volume ($V_{0}$ (V)), current (I (A)), electrical resistance (R (Ω)), pulse duration ($t_{d}$ (s)), electrical resistivity (ρ (Ωmm)), initial cross-sectional area ($A_{0}$ (mm^2^)), and initial length ($l_{0}$ (mm)). The electric energy density can be calculated with the nominal current density ($I/A_{0}$ (A/mm^2^)), electrical resistivity and pulse duration. In this paper, the electrical resistivity of 10^−4^ Ωmm is utilized for a general steel sheet. The calculated energy densities are listed in Table 4 to be corresponding to each electric current condition. Figure 5c shows the springback reduction regarding the electric energy density. The expression of (100 A 0.50 s) means the nominal current density of 100 A/mm^2^ with the pulse duration of 0.50 s. Since the electric energy densities of (50 A 0.50 s) and (75 A 0.25 s) are similar to each other, the springback angles are almost the same with the amount of about 31°. In the case of (100 A 0.50 s), the corresponding energy density is the maximum, so the springback reduction is the largest with the amount of 85.4%.

**Figure 6:**
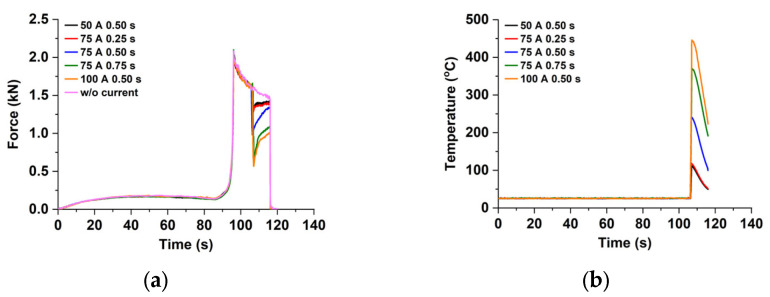
Responses with respect to the time during the V-bending test: (**a**) Force; (**b**) Temperature.

In order to support the macroscopic observation, the force and the temperature with respect to the time curves are investigated, as shown in Figure 6. Figure 6a shows the force‒time curves with various electric currents. When the punch, the specimen, and the die are contacted by each other, the force level rapidly increases. It decays during the holding position of the punch due to stress relaxation. The force relaxation is observed for all conditions while the force level suddenly drops the onset of applying the single-pulsed current. The force then recovers to a certain value during the holding since the electric current is released from the specimen. The amount of the force drops and the force level just before the unloading vary with the electric current conditions. The amount of the drop increases as the electric energy density increases while that of the force level just before the unloading decreases. From the comparison between the force just before the unloading for cases without current and the (100 A 0.50 s), 1.47 kN and 1.01 kN, respectively, it is noted that the electric current delays the force recovery and induces the force relaxation. The temperature histories with various electric currents are depicted in Figure 6b. The temperature suddenly increases to a peak value onset of applying the electric current due to the Joule heating. It dramatically decreases since the current is released. The peak value and temperature just before the unloading increase as the electric energy density increases. The temperatures just before the unloading are 49.4 °C and 223.0 °C for the largest (100 A 0.50 s) and the smallest (50 A 0.50 s) energy density cases, respectively.

## 4. Discussion

For the springback reduction, the single-pulsed electric current with a high electric energy density is remarkably effective. It seems that there exist both thermal and athermal effects on the force relaxation, which leads to the reduction of the springback. In this section, force levels from the experiment and prediction based on only the thermal effect are compared to clarify the dominance of each effect. The applicability of the electrically single-pulsed current into sheet metal forming processes is also discussed through the investigation of the microstructures and the hardness of the deformed specimens.

### 4.1. Effect of Electrically Single-Pulsed Current

**Figure 7:**
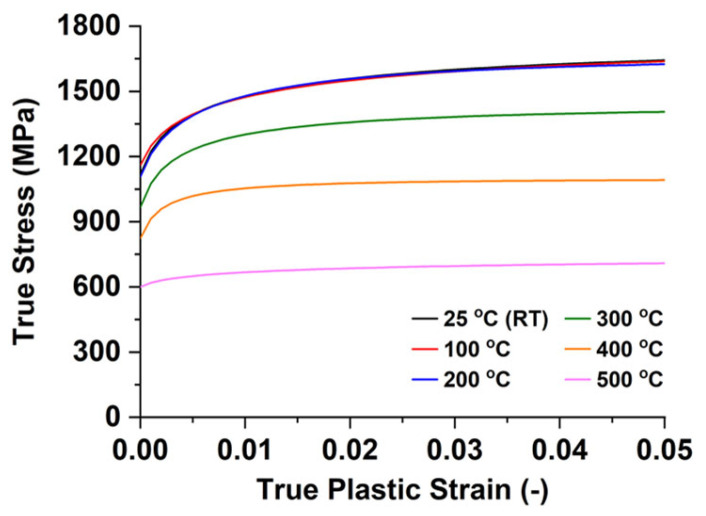
True stress‒strain curves at various temperature.

In order to confirm the thermal effect from the Joule heating on the flow stress, isothermal tensile tests were performed by using the MTS810 with the environmental heating chamber. Tensile specimens were prepared from the sheet following the ASTM E8 sub-sized dimensions (6 mm gauge width and 25 mm gauge length). Tests were conducted at 25 °C (room temperature), 100 °C, 200 °C, 300 °C, 400 °C, and 500 °C. The temperature range from 25 °C to 500 °C was determined based on the temperature levels that specimens experienced during the V-bending tests with the single-pulsed current shown in Figure 6b. The strain data were measured with a 3D digital image correlation (DIC) of the ARAMIS 12M system. The target strain rate for tensile tests was designed with the quasi-static value of 0.001 s^−1^. Figure 7 shows the true stress‒strain curves in tension at various temperatures. From the room temperature (RT) to 200 °C, the flow stress level does not change. It, however, decreases noticeably as the temperature increases from 300 °C to 500 °C. The thermal softening behavior is frequently observed in ultra-high-strength martensitic steels because phase partition and transformation start at a higher temperature [22,23,24].

In order to evaluate the effect of the electric current, the force and the temperature in Figure 6 were considered at the point just before the unloading. When the thermal effect by Joule heating exists only, the dropped force due to the electric current will recover to a value that is proportional to the flow stress level at the temperature just before the unloading. For simplicity, a basic assumption is that the force just before the unloading is independent of the temperature history but dependent on the temperature just before the unloading. Two concepts of ratios: (a) force ratio; (b) stress ratio, are defined by Equations (4) and (5) to clarify the effect of electric current and that of Joule heating.

(4) $$\mathrm{Force}\;\mathrm{ratio}\;(\%)=\left|1-\frac{F_{\mathrm{ref}}-F}{F_{\mathrm{ref}}}\right|\times 100$$

(5) $$\mathrm{Stress}\;\mathrm{ratio}\;(\%)=\left|1-\frac{\sigma_{\mathrm{ref}}-\sigma }{\sigma_{\mathrm{ref}}}\right|\times 100$$

**Figure 8:**
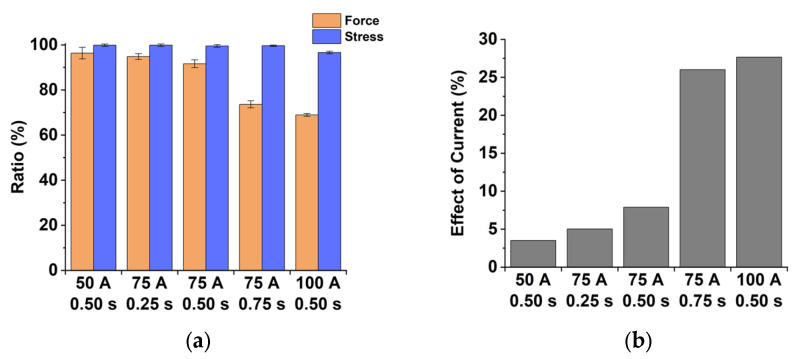
Force ratio, stress ratio, and effect of current regarding current conditions: (**a**) Force and stress ratios; (**b**) Effect of current.

The force without current ($F_{\mathrm{ref}}$ (kN)) and that with the electric current (F (kN)) are obtained from the force‒time curves at the last point (just before the unloading). The flow stress at room temperature ($\sigma_{\mathrm{ref}}$ (MPa)) and that at the temperature just before the unloading (σ (MPa)) are estimated by interpolation of the flow stress data in isothermal tensile tests. The stress values corresponding to the maximum bending strain (ε = 0.029) are determined to represent the stress just before the unloading. The maximum bending strain can be calculated by $\varepsilon =\ln\left(1\pm \frac{t}{2R_{i}+t}\right)$ where the $R_{i}$ is inner radius, i.e., punch radius and t is the thickness of specimen [25]. These two ratios are expected to be identical since the bending force is the first-order proportional to the flow stress [25]. Figure 8 depicts the force ratio, the stress ratio, and the effect of electric current regarding each condition. The error bars indicate the standard deviation of force and stress ratios. In Figure 8a, the difference between the force and the stress ratios can be understood as the effect of electric current because the difference means the amount of the force relaxation due to the electric current. Figure 8b shows the effect of the electric current calculated from the difference between average values of the force and the stress ratios with respect to the current condition. It is noted that the dominance of the electric current effect increases when the electric energy density increases. In the cases of (50 A 0.50 s) and (100 A 0.50 s), the values of the electric current effect are 3.5% and 27.7%, respectively. The springback reduction can be explained by the force relaxation due to the single-pulsed current.

### 4.2. Applicability of Electrically Single-Pulsed Current

**Figure 9:**
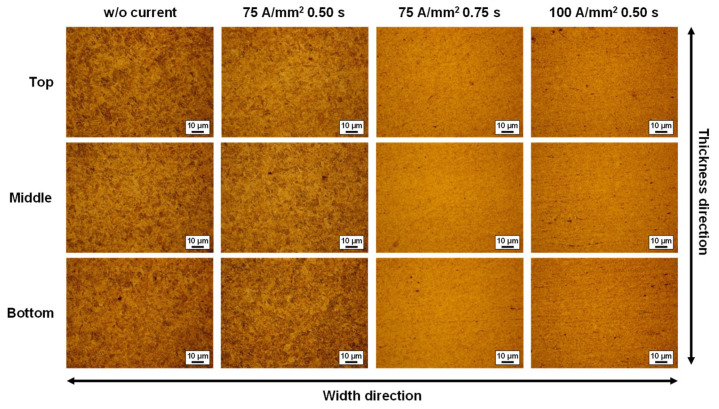
Microstructural observations from the V-bent samples.

To briefly evaluate the changes in material characteristics after applying the single-pulsed current, the microstructure and the Vickers hardness were obtained from the sample at the center of the V-bent specimen. The testing conditions of w/o current, (75 A 0.50 s), (75 A 0.75 s), and (100 A 0.50 s) were selected to investigate remarkable changes due to the electric current because the springback reduction with the electric currents was significant. The samples from the center of the V-bent specimens were mechanically cut and polished by using emery papers first. Those were then etched by a solution containing the perchloric acid of 50 mL and the methanol of 950 mL. Figure 9 shows the microstructure images obtained from an optical microscope (OM), ECLIPSE MA200, with a magnification of 1000. The microstructures were observed at three points: the inner layer (top), near-neutral layer (middle), and outer layer (bottom). A remarkable difference between cases of w/o current and (75 A 0.50 s) while (75 A 0.75 s) and (100 A 0.50 s) show totally different microstructures from the w/o current case. It is noted from the results that microstructural evolution would be observed when the applied electric energy is high. However, the springback is still reduced even though the microstructure does not change the (75 A 0.50 s) case likewise. For a better understanding of the microstructural evolution, there needs to be further microstructural studies, including the observations of SEM or TEM images.

**Figure 10:**
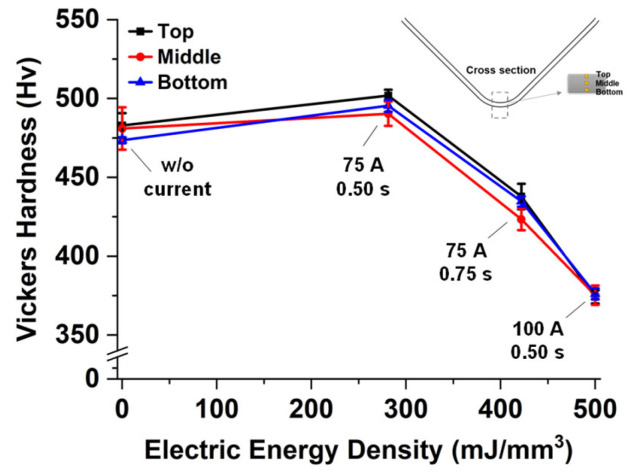
Measured hardness regarding the electric energy density.

To identify changes in the strength after imposing the electric current, micro-Vickers hardness tests were performed by utilizing a micro-hardness testing machine of the Mitutoyo 810–127 K. Likewise, the hardness measurement was conducted from the three points of the top, middle, and bottom, meaning the inner, near the neutral and the outer layers respectively. Figure 10 shows the measured hardness with standard deviations (error bar) regarding the electric energy density. The hardness in the case of w/o current is comparable to that in the case of (75 A 0.50 s). In the cases of (75 A 0.75 s) and (100 A 0.50 s), however, the hardness differs from the w/o current case and decreases as the electric energy density increases. It reveals that flow stress would decrease due to high electric energy, which might be an undesired situation for manufacturing structural components. The observations that the hardness remarkably drops at high electric energy while the springback is reduced even at lower electric energy are in agreement with the previous work [24] on the springback and the Vickers hardness changes of ultra-high-strength steels by the Joule heating. The tendency of the hardness corresponds to the microstructural observations. Because the microstructural does not change remarkably, the hardness values are comparable for the cases of w/o current and (75 A 0.50 s). On the other hand, the hardness values for both cases of (75 A 0.75 s) and (100 A 0.50 s) are decreased as well as far from the reference value of w/o current case since the microstructural evolution is observed. Therefore, it is noted that the single-pulsed current with a proper parameter set is very effective and useful to reduce the amount of the springback without changes in material properties. When the main purpose is the springback reduction regardless of the property changes, higher electric energy would be a good solution to attain a huge amount of the springback reduction.

## 5. Conclusions

The reduction of springback by an electrically single-pulsed current is investigated through the V-bending tests for the ultra-high-strength martensitic steel sheet of MART1470 1.2t. To perform the V-bending tests with various electric currents, a ceramic V-bending punch and die are fabricated to insulate themselves. It is found from the experiments that the springback was reduced with an increase in the current density, the pulse duration, and the electric energy density. The amount of springback reduction is 85.4%, with an electric current density of 100 A/mm^2^ and a pulse duration of 0.5 s. The springback reduction is caused by the force relaxation due to the single-pulsed current applied. To clarify the thermal and the athermal effects, analysis of the force level just before the unloading was performed based on the isothermal flow stresses in tension at temperatures from 25 °C (RT) to 500 °C. The dominance of the electric current effect increases as the electric energy density increases. Related to the applicability of the single-pulsed current in the sheet metal forming process, the microstructural images, and the Vickers hardness were obtained to confirm the changes in material characteristics. It is noted that the single-pulsed current with a proper parameter-set (75 A/mm^2^, 0.5 s) is effective in reducing the amount of the springback up to 37.5% without changes in material properties. When the main purpose is the springback reduction regardless of the property changes, higher electric energy corresponding to (100 A/mm^2^, 0.5 s in this work) would be a good way to reduce the springback.

## Figures and Tables

**Table 1:** Chemical composition of the MART1470 1.2t.

Chemical Composition (wt%)									
C	Si	Mn	Cr + Mo	B	Al	S	P	Nb + Ti	Cu
0.28	0.40	1.30	1.00	0.01	0.01	0.01	0.02	0.10	0.20

**Table 2:** Parameters of the Swift hardening equation.

$\mathrm{Swift}\;\mathrm{Hardening}\;\mathrm{Equation}\;\sigma \;=\;k{\left(\varepsilon_{0}\;+\;\varepsilon \right)}^{n}$		
k	$\varepsilon_{0}$	n
2176.6	0.0007	0.0889

**Table 3:** Testing conditions of the single-pulsed current.

Testing	Single-Pulsed Current		
	Applied Intensity (A)	Current Density (A/mm^2^)	Pulse Duration (s)
w/o current	-	-	-
Effect of current density	360	50	0.50
	540	75	
	720	100	
Effect of pulse duration	540	75	0.25
			0.50
			0.75

**Table 4:** Springback after V-bending tests with the electric current.

Current Density (A/mm^2^)	Pulse Duration (s)	Electric Energy Density (mJ/mm^3^)	Springback Angle (°)	Springback Reduction (%)
w/o current		0	34.06 ± 1.19	-
50	0.50	125.0	31.61 ± 1.04	7.2 ± 3.0
75	0.25	140.6	31.34 ± 0.43	8.0 ± 1.3
75	0.50	281.3	21.28 ± 0.55	37.5 ± 1.6
75	0.75	421.9	10.89 ± 0.59	68.0 ± 1.7
100	0.50	500.0	4.96 ± 0.16	85.4 ± 0.5

## Data Availability

The data presented in this study are available on request from the corresponding author.
